# The Israeli Rett Syndrome Center. Evaluation and Transdisciplinary Play-Based Assessment

**DOI:** 10.1100/tsw.2006.198

**Published:** 2006-10-10

**Authors:** Meir Lotan, Iris Manor-Binyamini, Cochavit Elefant, Judy Wine, Einat Saraf, Yael Yoshei

**Affiliations:** Israel Rett Center, National Evaluation Team, Chaim Sheba Medical Center, Tel HaShomer, Ramat Gan, Israel

**Keywords:** Rett syndrome, assessment, teamwork, play-based assessment, multiple disabilities, evaluation, Israel

## Abstract

Rett syndrome (RS) is a neuro-developmental syndrome of genetic origin, which mainly affects women. Individuals diagnosed with RS exhibit a variety of functional difficulties, which impair their quality of life. The variety of impairments and the differences between each child makes it necessary to administer skilled treatment, individually tailored to each client. Since the foundation of proper treatment is based on a structured, well administered, insightful assessment, the individual with RS with her complex array of difficulties should benefit from such a procedure. This notion has led to the establishment of the Israel Rett Syndrome Center. The center includes a medical branch located at the Safra Shildren's Medical Center at Tel Hashomer and an education/rehabilitation team, who performs assessments in special education facilities and residential settings throughout Israel. The assessment team works by means of arena assessment according to the concept of play-based assessment. This article presents the working model used by the education/rehabilitation team at the Israeli Rett Syndrome Center. The principles and working characteristics of the Israel Rett Syndrome Center team are suggested here as a potential model for establishing additional teams, presenting similar evaluation services for other individuals with RS as well as for analogous populations.

## INTRODUCTION

Rett syndrome (RS) is a genetic disorder that primarily affects females[1,2]. Approximately 1 of every 10,000 females will develop RS, a severely disabling neurodevelopmental disease[3]. Individuals with RS present an array of versatile phenotypes. Patients with more severe phenotypes might show early symptomatology without the period of normal development, and have congenital hypotonia and infantile spasms. Patients with a milder variant may retain some speech and motor functions without seizures[4,5,6]. This phenotypic variability is mainly the result of different mutation types and locations and distinct patterns of X chromosome inactivation[7]. The phenotypic versatility presented by individuals with RS necessitates a specific, knowledgeable evaluation as the cornerstone for a proper management program to be administered for this population. In order to assess the proper model for the evaluation of individuals with RS, one must perceive the past processes and existing models of evaluation for individuals with multiple disabilities.

## DEVELOPMENT OF EVALUATION MODELS

The concept that integrated teamwork can enhance the efficacy of services delivered to individuals with multiple disabilities is not new. This idea was first proposed by Whitehouse in 1951[in 8]. Today, more than 50 years later, paraprofessional therapists still find it difficult to be involved in integrated-type treatment, mostly due to the fact that each member of the educational/therapeutic staff was trained to work as an independent, therapeutic provider[9].

The variety of physical, cognitive, therapeutic, educational, and mental needs of patients with developmental disabilities presents challenges to the paraprofessionals responsible for their treatment. Accepting this point of view has led support services for pupils with special educational needs to change drastically during the past few decades. Today, strong pressure is being exerted by support services to develop cooperative transdisciplinary teamwork. In many countries such as Australia, England, the U.S., France, and Holland, teamwork has become the guiding component for establishing the policies of education and welfare services. This, in turn, has led to comprehensive changes, such as the development of cooperative assessment by a number of support services. In a broader sense, this process has also led to legislation obligating cooperation between different support services[10]. The rationale behind this policy is based on the fact that solving “complex problems” demands a wider scope of knowledge and more advanced capabilities usually not achievable by a single therapist[11,12]. In actuality, this policy has brought about a process of change and a transition from treatment methods based on the medical model (such as the multidisciplinary and interdisciplinary approaches) to methods such as the transdisciplinary model better suited to the educational environment. The transdisciplinary model was first developed in 1976 by the United Cerebral Palsy Association’s service for infants with cerebral palsy. After its assimilation, this working model was recognized by professionals in other therapeutic milieu, such as occupational therapy[13], physiotherapy[14], special education[15], nursing[16], medicine[17], and rehabilitation advisors[18].

This model was also recommended as a preferred approach for treating populations with multiple disabilities by care providers’ organizations in the U.S., such as ASHA (American Speech-Language-Hearing Association), AOTA (American Occupational Therapy Association), APTA (American Physical Therapy Association), and TASH (The Association for Persons with Severe Handicaps). Youngson-Reilly et al.[18] described three types of transdisciplinary teams whose objectives invite cooperation: application teams, discussion teams, and assessment teams. The present article deals with an assessment team (to find more on transdisciplinary assessment, see Linder[19]), providing services for individuals with RS.

RS is a severe, neurodevelopmental syndrome, characterized by a cessation of brain development[20], by loss of manual function and speech abilities, as well as cognitive, communicative, social, behavioral, and functional disabilities. These disabilities first appear at age 6–18 months, following an initial period of apparently normal development[3]. RS occurs in 1 out of 10,000 female births[3] and is the second-most common syndrome among women, the first being Down syndrome[21]. The individual with RS shows a complex clinical picture necessitating an evaluation by a transdisciplinary type of intervention. As of today, there is no known medical prevention or cure for RS, but effective, supportive treatment can extenuate the symptoms that characterize the syndrome[22].

The numerous and varied educational and therapeutic needs of girls with RS, together with their relative longevity, around 50 years[23], as compared to other syndromes of similar severity, demand a professional, transdisciplinary treatment. RS presents numerous and complex difficulties for the individuals and their families. Unfortunately, girls with RS and their families do not always receive the best possible treatment[24]. In light of this, there seems to be a need to adapt and improve management programs for individuals with RS and their families. The Israeli model was built under such circumstances with the aim of coping with the wide range of difficulties presented by this syndrome.

## THE ISRAELI MODEL

In 2003, the association known as the Israel Rett Syndrome Center (IRSC) was established and managed by families of girls with RS. The umbrella goal of the IRSC was to improve the quality of life for individuals with RS and their families. This goal should be attained through several routes:

- Promoting awareness of RS among the general public and among the medical and paraprofessional community, in view of the syndrome's rarity
- Providing support and counseling for parents of individuals with RS
- Assessing individuals with RS by means of a team of experts
- Providing guidance for educators and therapists working with individuals with RS
- Promoting and funding research on RS[25]

Two structures were established for this purpose: the Rett Syndrome Medical Clinic at the Safra Children's Hospital, Sheba Medical Center at Tel-Hashomer and the rehabilitation/education assessment and counseling group of the IRSC.

### The Rett Clinic

The objectives of the RS clinic was to provide comprehensive services for individuals with RS and their families from the medical, therapeutic, and educational standpoints, while taking the unique characteristics and needs of each child into consideration (The IRSC provides group meetings for parents that are carried out within a separate framework, outside the scope of the present article). These services are aimed at helping the child to attain optimal function. Diagnosis and assessment at the facility include professional guidance such as:

- Counseling for the family and other supportive frameworks
- Recommendations and guidance for a holistic treatment program within the community framework
- Follow-up of the condition and treatment program for each child

#### *Description of Activities*

Since the present article mainly focuses on the Center's paraprofessional assessment and guidance team, the activities of the medical arm of the RS clinic will only be briefly described here.

The Rett Syndrome Medical Clinic treats all girls in Israel with RS who have been located (as of today there are approximately 100 known individuals with RS in Israel). The clinic is located in the Sheba Medical Center, Tel Hashomer and directed by a senior pediatric neurologist. The team includes an orthopedic surgeon, a gastroenterologist, an endocrinologist, a geneticist, and a dietician. During each medical evaluation, the child and her parents meet with the head of the team, who examines the individual with RS, and provides the parents with information regarding the syndrome and the child's expected prognosis and direction for intervention according to the each client's needs. Each child undergoes orthopedic, gastroenterological, and dietetic examination and evaluation. If the child has not yet undergone a genetic diagnosis, she is also referred to a geneticist for genetic advising and evaluation. Due to the erratic nature of RS, the family is encouraged to contact the head of the medical team by phone or e-mail, and to seek counseling in dealing with daily difficulties or emergency situations that might arise. The RS clinic continues to follow each child's progress on an annual or semiannual basis, depending on the age of the child and the severity of her phenotypic expression.

## The Evaluation and Guidance Center

The Evaluation and Guidance Team was established with the objective of assessing girls with RS and for counseling the therapeutic, educational, and rehabilitation staff, who supports the individual with RS on a daily basis.

The Rett Center assessment team is a transdisciplinary team consisting of a special education teacher/advisor, a music therapist, a speech therapist, an occupational therapist, and a physiotherapist. The professional aim of this team is to conduct assessment and advisory services for each child within her special education facility in the presence of the educational staff and the family. In certain cases, such as when the child is too small to be integrated into a kindergarten or is in transition between special education facilities, assessment and advising are performed at the Tel Hashomer clinic. All of the assessments are fully funded by the IRSC. The Rett Center team has developed a unique assessment model that provides an answer to the comprehensive needs of girls with RS. The structure of the model is described below.

### *Assessment Structure*

#### A. Pre-Evaluation

Weeks before carrying out the meeting in the educational facility, a summary of the child's evaluation at the Tel Hashomer clinic is sent to all team members. Preliminary working contacts are established between the RS team coordinator, the parents, and the educational facility staff. This period of time enables an exchange of information and expectations.

#### B. Assessment

The assessment itself is a fluent, ever-changing, process (see Fig. 1), but for the purpose of simplification, the model is presented here in more detail and in a much more structured way than it is actually performed.

The first objective is to get acquainted with the educational team and present the objectives of the meeting. At the day of the evaluation, before the assessment begins, a short meeting is held with the educational/therapeutic staff of the child's special education facility and the parents. During this meeting, expectations are clarified and missing information regarding the child's function illuminated by the educational/therapeutic staff. In light of the objective difficulties that arise, when an alien team is introduced into a special education or rehabilitation facility, this stage must be carried out with the proper attention devoted to details[26], yet without losing sensitivity. This stage enables the members of the RS center team and the members of the staff of the special education facility to become acquainted. Sufficient time is allotted for constructing preliminary cooperative working connections and agreeing on objectives that suit the expectations of the parents and the educational/therapeutic staff.

Thereafter the RS team initiates contact with the individual with RS. Since girls with RS have difficulty adapting to unfamiliar people and situations[27,28,29], the first encounter takes place between the child and the music therapist, who serves as a moderator involving the child with the assessment team.

After the first encounter, the assessment will take place according to the "play-based assessment" model. During the assessment, various members of the team direct the proceedings and engage in activities with the child, by means of mediation through the music therapist, or by joining in on the activity. The special emotional bond that girls with RS have with music is a known phenomenon that has been described at length in research literature[27,29,30,31,32,33,34]. In light of this special love held for music by individuals with RS, it is the optimal therapeutic tool for attaining maximum function and cooperation. Therefore, the music therapist is the front person to establish primary connection with the child.

## C. Summary

At the termination of the assessment with the child, a summary discussion is held where the major findings of the assessment team are clarified and the main suggested directions for intervention with the child are summarized.

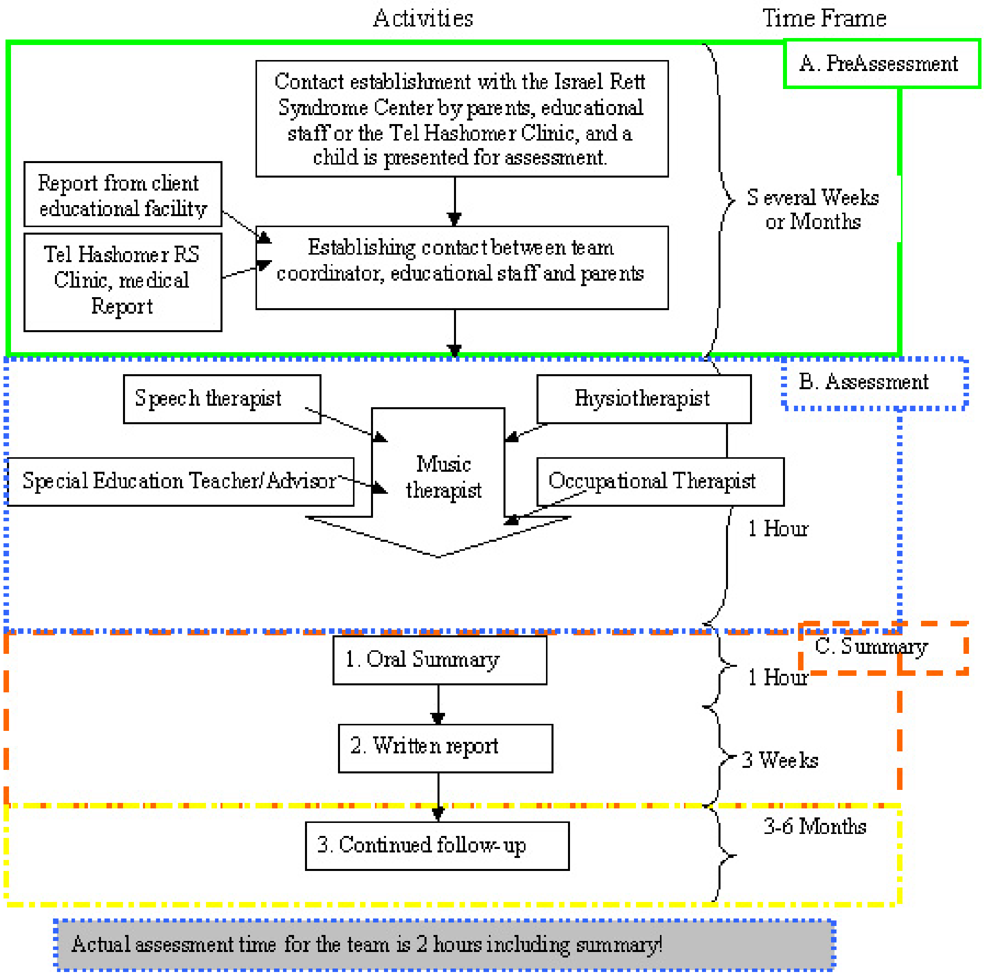

**FIGURE 1.** Chronological flowchart for performance of assessment for individuals with RS.

Several weeks after the assessment, a summary report is issued to the parents and educational/therapeutic facility, which includes the main suggestions presented at the meeting, but with a generalization of clinical recommendations and elaboration regarding intervention options.

## D. Continued follow-up.

In some cases, an additional follow-up meeting is held between the Rett Center assessment team and the educational/therapeutic staff of the special education facility, 3–6 months following the presentation of the written summary report. During this meeting, the clarity of the recommendations is verified and the applicability of the suggestions within the daily routine of the special education facility evaluated. If problems have arisen in the process of applying the recommendations, common efforts are investigated to solve those problems. These follow-up meetings do not always take place due to budgetary concerns (and due to the fact that they are not always necessary), but they are an important component of the model.

## *Fundamental Aspects of the Teamwork*

In order for this model to work and for readers to sense the complexity of this approach, the guidelines according to which the assessment team works are laid out. These guidelines have been assembled by the assessment team of the IRSC and are constantly being revised and developed.

### Accessibility

- **Physical accessibility**. Since the child spends most of her day in the special education facility, it is perceived as the child’s natural surroundings and the assessment is therefore carried out there. Exceptions for visiting the girls’ educational facilities are in cases were the child is still too young to be integrated into a special education facility, or when there is an urgent request from the parents to see the child during a transition phase between educational frameworks.
- **Online accessibility**. The RS Center staff is accessible by telephone or e-mail to both parents and the educational/therapeutic staff.
- **Financial accessibility**. Assessment is fully funded by the Israeli Rett Syndrome Center (IRSC).

### Family Directed Assessment

Assessment usually takes place following a request from the parents of the child with RS to the RS Center or following a request by the staff of the special education facility. Before the assessment begins, the parents or educational/therapeutic staff clarifies the objectives and expectations of the meeting. Meetings are usually held at the child’s special education center. In several cases, special consideration requires that the place and character of the meeting will be adapted to each family and child according to their objectives and expectations (see Table 1).

**TABLE 1**
**Assessment Location and Objectives as Defined by the Child’s Parents**

| Child’s Name | Location | Objectives Defined by Parents |
|---|---|---|
| A.B.H. | School | Offering feedback and points of emphasis for the child’s Individual Educational Program (I.E.P) |
| A.E. | Child’s home | Offering activities and promoting motor skills and communication in the framework of the home |
| G.O. | Tel Hashomer Hospital (Rett Clinic) | Initial directions for activities and assisting the parents in locating and organizing the management program for the child (a young child recently diagnosed with RS) |

## Coordination

Each assessment meeting is meticulously coordinated between the coordinator of the RS team, the parents, and the staff of the special education facility. For each child, previous medical, educational, and developmental assessments are sent to the members of the RS team. After using those past reports to familiarize themselves with the child's state, the framework and type of meeting are agreed ahead of time according to the unique needs of the child, the special education facility, and the parents.

## Flexibility

Because of the different character, structure, and needs of each child and her parents and the differences between various special education facilities, each meeting begins and progresses in a different manner. The IRSC team adapts itself to these differences in order to offer an appropriate response to each special education facility within any forum. Sometimes assessments begin with a short lecture about RS. In other cases, the staff of the educational center presents the work they are doing with the child, while the RS team observes their work and suggests possibilities for improvements. In other cases, a specific point is raised by the staff or parents as being problematic with a specific child, such as exercise, intervention using the Snoezelen, activities in the therapeutic pool, communication, music therapy, etc. In these cases, advice is focused on the specific problem. When it is deemed necessary, individual intradisciplinary meetings are held.

## Comprehensiveness

Our ability to supply comprehensive assessments stems from the cooperation between team members who are participating and from the type of assessment that is designed to account for the variability of needs, wishes, and abilities of the child, the parents, and the special education facility in consideration.

Each assessment/advising meeting is usually held with all members of the paramedical team in the form of transdisciplinary assessment. Since the meetings are adjusted to meet the needs of the client, the parents, and the educational facility, they are flexible in nature. Meetings sometimes takes place with only one or two team members, if such counseling is requested in a specific educational/therapeutic domain.

Assessment is performed by means of play-based assessment methods. Play-based assessment is a part of the transdisciplinary approach[8,35,36]. This approach recognizes common responsibilities and the need for teamwork among paraprofessionals involved in intervening with children with different disabilities and their families. During these assessment meetings, the child interacts with one (usually the music therapist) or more moderators (sometimes the child's parents, teacher, occupational therapist, or team member are involved) and the rest of the team members observe and are actively involved in the interaction among the child and the moderator. This approach is a more global method of assessment, and is carried out in a calm and informal atmosphere in which the child can feel as relaxed as possible[18].

The aim of this comprehensive assessment method is for parents and members of the educational/therapeutic staff to participate. Our professional opinion is that an individual with RS will function at her best when she feels included and she senses she is fully accepted, supported, and contained through empathy. The central lead in the assessment team is usually the music therapist, who adjusts her approach to each child's emotional situation and level of communication. Initial contact is usually achieved through the use of live music, such as familiar songs, or improvised modes of communication. The contact initiated by the music therapist provides the child with a protected and secure framework that enables her to enjoy herself and cooperate to the fullest. An additional member of the Rett Center team, usually the physiotherapist, holds the child and provides physical support and mobility when needed (see Fig. 2). After the establishment of good rapport and trust by the child, other activities are added to the musical activities according to the objectives of the assessment team. During the entire assessment, other members of the team observe and are actively involved in the interaction.

For example, if a problem in handling food was predetermined or observed during the assessment, the occupational (OT) and speech therapist (ST) will lead the evaluation process at occasions when such intervention is warranted. In addition, information regarding the child's capabilities is constantly collected from the educational/therapeutic staff and the parents, and is immediately integrated in to the evaluation process. The team coordinator narrates the meeting, mainly to the parents, regarding what actual goal and interactions are applied, in real time.

In cases were the educational staff members' requests that intraprofessional meetings will be held, each member of the RS Center team holds a small-scale gathering with his corresponding discipline member of the educational/therapeutic staff.

It is important to reiterate that the formal written description of the model is very structured and presented pragmatically. In actuality, however, there is a constant flexible flow that enables spontaneous assessment in a friendly atmosphere. This is important to promote cooperation and to adapt the assessment process to the needs of the child, family, and educational staff. Such an atmosphere has direct impact on the degree to which we can become acquainted with the child, the level of trust that is established, and the degree of openness that is attained.

**Figure 2 – Evaluation model**

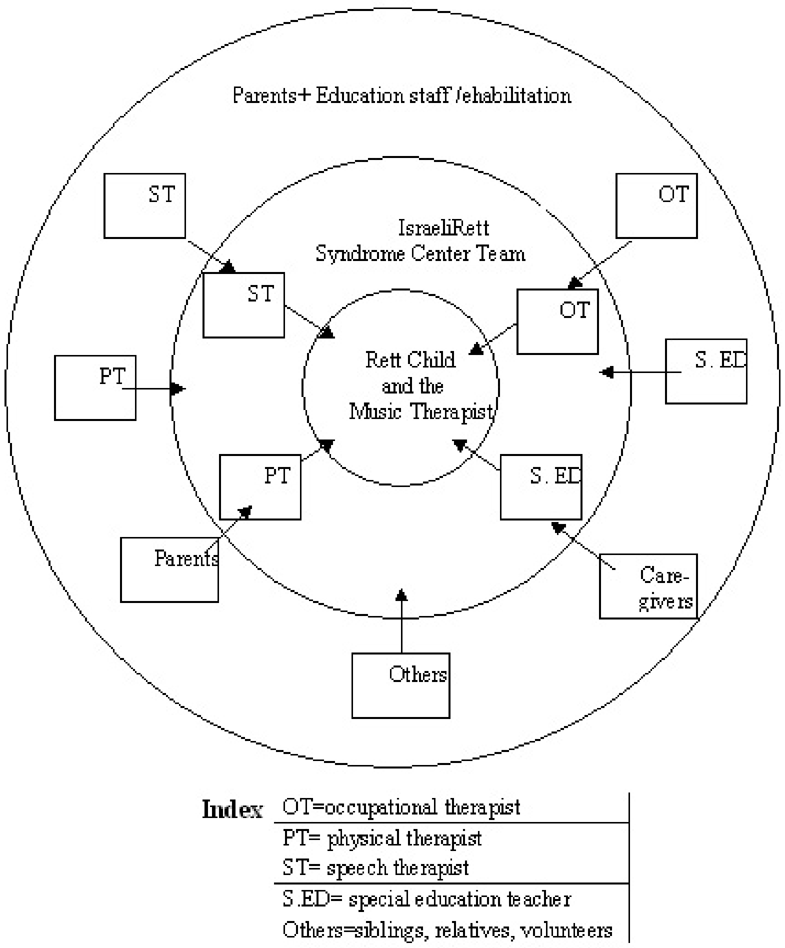

**FIGURE 2**. Evaluation model.

### Continuity

Assessment continuity is ensured by adhering to the following elements. A detailed report is issued 3–4 weeks following the meeting with the child, her parents, and the educational staff. This report is sent to the parents and educational staff and includes the findings of the transdisciplinary team. A copy of the report is also added to the child's medical records at the Sheba Center at Tel-Hashomer. This report emphasizes concrete clinical and educational recommendations. Furthermore, the family can receive a video film taken during the assessment. The movie can also be viewed by the educational/therapeutic staff. A follow-up meeting is held several months after the initial assessment and after the recommendations suggested by the RS team have been applied by the educational staff. The RS team meets again with the child's parents and educational/therapeutic staff. Such a meeting is designed to answer any questions that remain unanswered, to clarify findings that were presented during the previous meeting, and to jointly brainstorm regarding difficulties that have arisen during the application of the recommendations.

### Follow-Up and Feedback

Following the evaluation of the RS team, a feedback form is given to the parents and to members of the educational team. This form evaluates the efficiency of the performance of the RS team and enables the IRSC to supervise the work of the team. The members of the RS team are also required to fill out a self-evaluation form regarding every meeting in order to learn and draw conclusions regarding their function. The degree of compatibility between the answers that the RS team provided and the ones filled by the parents/educational facility team enables a cross-correlation of the efficacy of the team and improves the quality of the service that it offered. In this manner, the team can improve its work in future meetings.

## *Intervention Methods*

The assessment team of the IRSC employs several intervention approaches:

- **Assessment**. The most common method is assessment of the individual with RS at the request of the parents or educational staff. This method was fully described in the previous section. However, there are instances in which different forms of intervention are needed. In these cases, the team is prepared to provide solutions in other areas, such as consultation or problem solving.
- **Consultation**. Since the members of the team have comprehensive knowledge regarding developmental syndromes, parents (particularly young parents) can be given guidance in various subjects, such as suitable special education facilities near their place of residence, agencies for renting equipment for the home or school, and therapists or care providers with knowledge about RS. (For example, the fact that equipment can be purchased from various suppliers with different price levels or, on the other hand, attained for free through different volunteer organizations, is not always known by the parents.)
- **Negotiations and problem solving**. When specific problems arise between parents and the child's educational facility, members of the team might be called upon to negotiate and bridge the gaps. In this way, the unbalanced system can return to a situation in which it provides agreed supportive care for the individual with RS in which she can progress. Examples of this type of intervention are:
    - **Case one**: A child was placed in a special education facility by the municipal education board, while the local educational team opposed the decision. The controversial decision resulted in opposition among the educational/therapeutic staff. This resulted in mistrust and severed relations between the child's parents and the educational/therapeutic facility. Following a meeting between the educational/therapeutic staff, the parents and the coordinator of the RS team, cooperation was restored between the staff and the family, and the child could once again receive proper care mainly due to restoration of the trust relationship between parents and the educational facility.

- **Case two**: Another example of this form of intervention was a case in which a child with RS fell and was slightly injured while exercising on a treadmill at her educational facility. After the incident, her parents decided to prevent her from taking part in any physical activity at the special education facility. The physiotherapist from the IRSC team met with the parents and the educational staff, and the result of this meeting was a mutual agreement on an exercise program that was shared between the home and the special education facility.

## DISCUSSION

The Israel Rett Syndrome Center (IRSC) administers a team of experts who work in a unique manner in accordance with the principles described in this article: Accessibility, family-directed assessment, coordination, continuity, flexibility, comprehensiveness, follow-up and feedback. These principles are designed to meet the needs of individuals with RS and their families, and to help paraprofessional teams working in special education facilities to acquire knowledge of the unique characteristics of individuals with RS in order to offer them the comprehensive care they require. The unique teamwork described in this article originates from a combination of the following factors:

- **Low cost**. The actual assessment according to the presented model takes only two hours in comparison to other countries in which the duration of the assessment takes about 5 days to one week.
- **Natural surroundings**. The RS team works by visiting the child's natural environment and by offering individual advice for the members of the educational staff who care for the child on a permanent basis.
- **Manner of assessment**. The evaluation of the client's condition is based on transdisciplinary play-based assessment (TPBA), which is an amiable form of assessment for the child and her parents.
- **Holistic manner of evaluation**. The entire IRSC team assesses the child's abilities and needs, and presents the educational staff and the parents with a complete therapeutic management program. Since the physical therapist is also acquainted with the Snoezelen approach and adapted sitting, and due to the fact that the occupational therapist is also a hydrotherapist, the team covers a variety of therapeutic fields suitable for this population.
- **The human factor**. Members of the team are characterized by a homogeneous perception of the treatment/education aspects regarding care for individuals with RS. In addition, all of the members of the Israeli team have been acquainted with and cared for individuals with RS for more than 15 years. This accumulated professional experience has enabled the team members to form professional identity. This is one of the critical elements that make the working framework described here possible.
- **Clinical approach**. The IRSC team supplies both the parents and the educational facility's staff with clinical, down-to-earth suggestions that are ready for implementation.
- **Feedback and follow-up**. A child who has undergone initial diagnosis and received treatment recommendations is visited again several months after the recommendations are first implemented for the purpose of feedback. This enables the construction of a feasible management plan for each child, which is acceptable and performable by the educational staff. All members of the RS team are kept fully accessible to the educational team and the parents via e-mail or by phone.

All the points mentioned above make the working methods of the IRSC team a unique model, both in Israel and, as far as we know, also abroad.

## CONCLUSIONS

The working model presented in this article is unique both in respect to the work methods used by the assessment team and in respect to the age (1–40) and the level of disability of the target population: children and adults with RS. Today, the work methods used by the assessment team are being explored more thoroughly in order to locate factors that will enable optimal use of the model and to continue to improve the team's working methods. The authors recommend that other national or regional centers adopt a similar working model in order to promote knowledge and improve the quality of services for individuals with RS.
